# Re-evaluating recurrence patterns and lymph node mapping in ICG-guided lymphadenectomy for gastric cancer

**DOI:** 10.1097/JS9.0000000000003103

**Published:** 2025-08-04

**Authors:** Xianjun Liang

**Affiliations:** Department General Surgery, Dalian Women and Children’s Medical Group, Dalian City, Liaoning Province, China

*Dear Editor,*

We read with great interest the article by Zhong *et al*[1] comparing submucosal (SMA) and subserosal (SSA) ICG injection techniques in gastric cancer surgery. The authors report comparable long-term outcomes between SSA and SMA, supporting SSA as a practical alternative. However, while the study includes recurrence as a secondary endpoint, it does not provide station-specific recurrence mapping or correlate lymph node failures with intraoperative fluorescence coverage. This limits the ability to evaluate the anatomical completeness of lymphatic dissection in regions with complex drainage patterns. We make sure our article is compliant with the TITAN Guidelines 2025, show in TITAN Guideline Checklist 2025[2].

ICG fluorescence-guided lymphadenectomy is an important advancement, enhancing lymph node yield and surgical safety. Yet, its oncologic utility fundamentally depends on whether the tracer reliably reflects true anatomical lymphatic drainage, especially in areas with complex or variable nodal basins. A 2022 meta-analysis involving 2274 patients demonstrated that ICG can increase nodal yield and reduce intraoperative blood loss without added risk[3]. However, the clinical benefit of ICG hinges not only on the quantity of nodes retrieved but on its anatomical precision in mapping lymphatic pathways relevant to tumor spread.

Numerous studies have established that gastric tumor location significantly determines lymphatic spread. For example, proximal tumors often metastasize to stations Nos. 1, 2, 7, and 9, while distal tumors more frequently involve stations Nos. 5, 6, and 8^[4,5]^. These patterns suggest that ICG injection efficacy cannot be universally assumed across all tumor subsites. Chen *et al*[6] reported that while both SMA and SSA injections are feasible, differences in tracer distribution can affect lymphatic mapping and the completeness of surgical dissection. This is especially relevant for SSA, where tracer spread relies on serosal lymphatics, which are less dense and potentially less predictable than submucosal pathways. As a result, SSA may inadequately label certain nodal basins, particularly in the upper gastric basins, when injected only from the anterior serosal surface.

Recurrence site distribution is closely linked to primary tumor location. For instance, proximal tumors have a higher risk of lung and bone metastases, while mid-to-distal tumors more frequently recur in the peritoneum or regional nodes[7]. Additionally, recurrence in specific nodal stations, such as station No. 6, has been associated with poorer outcomes and may define unique patterns of failure[8]. These anatomical recurrence trajectories suggest that blind spots may exist in SSA-guided lymphadenectomy, especially if fluorescence tracers do not adequately reach all critical nodal regions.

Beyond anatomical considerations, tumor biology, including histological subtype and molecular profiles, can influence patterns of spread and may affect how effectively ICG maps lymphatic pathways^[9,10]^. Although this area warrants further research, practical improvements such as station-specific fluorescence mapping during surgery and correlating fluorescence uptake with postoperative recurrence could enhance our understanding of SSA’s true oncologic safety.

While the FUGES-019 trial reports comparable overall recurrence rates between SSA and SMA, the absence of recurrence data stratified by lymph node station or tumor subsite limits the ability to assess whether SSA achieves truly equivalent lymphatic clearance across all gastric regions. Prior studies in post-D2 cohorts have shown that locoregional failures tend to follow predictable nodal pathways based on tumor location[11]. Without accounting for such anatomical specificity, SSA may appear equivalent overall but could still miss certain critical nodal basins in anatomically complex areas.

To improve confidence in SSA’s oncologic safety, future studies should incorporate station-specific recurrence mapping and correlate these data with intraoperative ICG fluorescence patterns, ideally supplemented by post hoc anatomical reconstruction. Moreover, integrating histologic subtype and key molecular markers into such analyses could further refine ICG-guided strategies, helping tailor lymphadenectomy to each tumor’s unique biology.

In conclusion, Zhong *et al*’s study advances fluorescence-guided gastric cancer surgery by validating SSA as a practical alternative to SMA. However, to fully establish SSA’s equivalence, further anatomical and potentially molecular refinements are needed. We encourage follow-up research that integrates recurrence topology, fluorescence quality metrics, and tumor characteristics to optimize precision and personalization in lymph node dissection for gastric cancer.

## Data Availability

Not applicable.
